# Influence Mechanism of Polycentric Spatial Structure on Urban Land Use Efficiency: A Moderated Mediation Model

**DOI:** 10.3390/ijerph192416478

**Published:** 2022-12-08

**Authors:** Di Zhu, Yinghong Wang, Shangui Peng, Fenglin Zhang

**Affiliations:** 1 School of Public Policy and Management, China University of Mining and Technology, Xuzhou 221116, China; 2 School of Environment Science and Spatial Informatics, China University of Mining and Technology, Xuzhou 221116, China; 3 School of Economics and Management, Shandong Agricultural University, Tai’ an 271018, China

**Keywords:** polycentric spatial structure, utilization efficiency of urban land, urban agglomeration, mediating effect, moderating effect, SBM

## Abstract

Under the background of green development, the spatial structure of urban agglomerations (UA) has an important impact on urban land use efficiency (ULUE), but few studies have explored the impact mechanism between the two. This research explores the impacts of polycentric development on ULUE of UA, using data for 140 cities in China’s top ten key UA covering the period from 2004–2019. The linkage between polycentric development and ULUE is explored by estimating models of determinants of ULUE. This research also examines the mechanism of the polycentric spatial structure of UA on ULUE by using a moderated mediation model. The main findings of the research can be concluded as below. The eastern UAs show a mostly polycentric spatial structure, whereas the central and western UAs show a weak polycentric spatial structure. The polycentric spatial structure of UA has a positive impact on ULUE. An inverted U-shape curve depicts the relationship between the polycentric spatial structure of UA and ULUE. However, the mediating variables, integration of industrial structure and factor mobility have a positive and partially mediating effect between the polycentric spatial structure of UA and ULUE. The infrastructure level has a positive U-shaped regulation effect, in which the impact coefficient of transport infrastructure is more significant. These findings provide empirical evidence for the coordinated development of China’s regional space planning and ULUE.

## 1. Introduction

As a core element of resources, policies, achievements and systems, land bears the track of urban construction and growth [1]. ULUE is a direct reflection of the coupling level between an urban system and a land use system. It is also a key indicator for measuring the rational allocation and efficient utilization of production factors under the background of high-quality development in China [2]. ULUE is directly related to regional sustainable development.

Therefore, improving the efficiency of urban land use and achieving sustainable regional development have become the consensus of all countries in the world. In the past few decades, China’s economy has grown rapidly, large- and medium-sized cities have expanded rapidly, and the level of urbanization has increased rapidly [3]. By 2022, China’s urbanization rate has reached 64.72%. As more and more people flock to large and medium-sized cities, the regional monocentric spatial structure patterns are gradually taking shape. This model is centered on large cities, and small and medium-sized cities with a low density of element agglomeration and connection are distributed around them. The monocentric spatial structure model brings products and elements closer to the core cities of the region, and the economy and life are highly concentrated in one core city within the region. However, the excessive concentration of resources also leads to a series of urban ills such as a mismatch between ULUE and the speed of regional economic development, the improvement of ecological environment lagging behind regional development and unreasonable regional land use planning [4]. There is growing evidence that high population agglomeration conflicts with limited land supplies [5]. As China’s economy has entered a stage of high-quality development, the expansion of large- and medium-sized cities in China has slowed down, and infrastructure and social security have been improved. Taking UA as the main body to build a new pattern of coordinated urban development has become a new requirement for China’s regional development and construction. In the new development stage, the economic distance and geographical distance between cities in the UA are increasingly shortened [6], and the regional economic development strategy is rapidly changing according to the regional coordinated development strategy. The spatial structure within the UA should be adjusted accordingly.

According to the development and planning experience of the world’s large UAs, the polycentric spatial structure is considered to be a more reasonable spatial structure in the mature stage of the development of large UAs [7]. Spatial structure is the distribution of population and economic activities in a certain region. It is one of the important determinants of resource utilization efficiency in a region. The polycentric spatial structure weakens the market boundary between cities, making the interactive flow of production factors and the spread of knowledge spillover more frequent [8]. It can provide significant channels for the industrial integration development of urban agglomeration and the flow of production factors [9,10]. In addition, the division of labor and cooperation between cities can produce a “1 + 1 > 2” convergence effect [11], and gradually break the market segmentation. The polycentric spatial structure facilitates the dissemination of resources, technology and even market information within the region. Therefore, the polycentric spatial structure can promote the systematic integration of land use and urban land classification [12]. On 24 February 2022, the State Council’s reply to the 14th “five-year” plan for the development of UAs clearly points out that the dynamic mechanism of regional polycentric development should be established. At the same time, research on the effects of economic agglomeration [13], industrial restructuring [14] and production factor input [15] on ULUE also demonstrates the close relationship between regional spatial structure and urban land use. Many scholars believe that the polycentric development of UAs can enhance regional economic competitiveness and environmental sustainability [16,17]. However, the reality is that in the process of advancing the polycentric spatial structure of UAs, the scale of land use of UAs is increasing, but the overall level of use and efficiency are still low [18]. The pattern of differential distribution within the UA has not fundamentally changed, and the collaborative performance between cities is not very obvious [19,20]. Similarly, some scholars have confirmed that the polycentric spatial structure of UA weakens the agglomeration economic effect and leads to a reduction in the utilization of production factors when the large cities have not yet reached the optimal scale [21]. Therefore, the following two questions have become the problems that China must face in the process of urbanization. Should the traditional model of regional single-center spatial structure development be abandoned? How to improve the level of urban land conservation and intensive use by optimizing the regional spatial structure?

For this purpose, this research uses 2004–2019 panel data of the top ten UAs in China and adopts the Rank-Size distribution-based approach and super-efficiency SBM-Malmquist model to measure the polycentric spatial structure and ULUE. The linkage between polycentric development and ULUE is explored by estimating models of determinants of ULUE. This research also examines the mechanism of the polycentric spatial structure on ULUE by using a moderated mediation model. The purpose of this paper is to provide relevant ideas and policy inspiration for China to promote the layout of regional polycentric spatial structure and improve the way of efficient and sustainable land use in the new period.

The remainder of this paper is structured as follows. Section 2 presents the literature review. Section 3 discusses the impact of the polycentric spatial structure of UAs on ULUE and develops the research hypothesis. The research design and related indicators are introduced in Section 4. We present the empirical analysis of the polycentric spatial structure of UAs on ULUE and the mechanism analysis in Section 5 and Section 6. Section 7 proposes the policy implications of this article based on the research conclusions.

## 2. Literature Review

The concept of land use efficiency originated from the Market Allocation Efficiency theory of neoclassical economics in the 1870s [22]. As urbanization grew, scholars began to focus on ULUE. For more than half a century, scholars have mainly studied the dynamic changes, evaluation and calculation methods and impact mechanisms of ULUE. In general, the literature in this area consists mainly of the following: (1) The changes of ULUE. Scholars have systematically studied the spatial and temporal characteristics [23], regional differences [24] and convergence characteristics [25] of ULUE on a multi-scale. The object of study included different types of land, such as urban agricultural land [26], forests [27] and arable land [28]. (2) Evaluation and measurement methods. The evaluation index of ULUE has been gradually shifted from a single index to a multi-index evaluation system with “economic-social-ecological” coupling [29]. As for the research methods, in recent years, scholars tended to choose the DEA model [30], super-efficient SBM model [31] and comprehensive index evaluation model [32] to calculate ULUE. (3) Impact mechanism analysis. Some scholars have analyzed the influencing mechanism of ULUE from the perspective of land market [33], housing prices [34] and land finance [35]. It can be found that urbanization level, openness to the outside world and land price are also key factors affecting ULUE [36,37]. In addition, some scholars discuss the correlation between ULUE and other systems from the perspective of coupling coordination. Regional Integration [24], ecosystem health [38] and urbanization [39] were included in the research objectives.

The concept of polycentric spatial structure derives mainly from urban spatial planning. With the development of the social economy, the polycentric spatial structure has been closely watched because of its important political and academic significance [40]. The current research mainly starts from the two paradigms of morphology and function to calculate the polycentric spatial structure [41]. On the one hand, scholars use the relevant data of population, night light or employment to calculate the Gini index and the Herfindahl index and Pareto index to quantify the measurement index of the polycentric spatial structure. On the other hand, scholars use capital factor flow and electronic communication levels to measure the polycentric spatial structure. With the gradual maturity of measurement methods, scholars gradually explore the influence of a polycentric spatial structure on energy efficiency [42], economic development [43], carbon emissions [44], etc.

At present, there are few direct research results on the influence of the polycentric spatial structure of UAs on ULUE. The related research is mainly divided into the following two categories. (1) From the perspective of urban spatial structure, scholars analyze and explore urban land use change. For example, Ding and Zhao [45] discussed the influence of the land market and property of housing production function on urban spatial structure. Dominguez et al. [46] modelled the spatial structure of Bogotá and 17 municipalities to analyze their land occupation and distribution of land use. (2) From the perspective of externality, some scholars discuss the relationship between urban expansion [47], economic growth [32], industrial upgrading [48] and ecological environment change [49] in the formation of a polycentric spatial structure.

All of this research is the indirect embodiment and practical basis of the correlation between a polycentric spatial structure and ULUE, but the following issues remain to be further discussed. First, current research on the impact of a polycentric spatial structure mainly focuses on economic development and is limited within cities. In the geographical scope beyond a single city, what impact will the polycentric spatial structure have on ULUE? Few scholars have conducted direct and systematic theoretical and empirical study on this issue. Second, some scholars tried to answer the first question and empirically demonstrated the impact of a polycentric spatial structure on ULUE based on UA data in China. However, they have not yet explored how the polycentric spatial structure will affect land use efficiency. The single panel model cannot fully explain the impact mechanism of a polycentric spatial structure on ULUE. Thirdly, as one of the conditions affecting the form of polycentric space, the level of infrastructure in UAs has a great impact on resource circulation, factor exchange and information transfer among cities within the agglomerations. Will the level of infrastructure have a moderating effect on the relationship between a polycentric spatial structure and ULUE?

Compared with the existing studies, the contributions of this paper are as follows. First, because the effect of spatial agglomeration on ULUE has already been studied, the impact of the spatial structure on ULUE is further investigated from a new perspective of the polycentric spatial structure in this paper. This paper also deeply analyzes the core influence mechanism of polycentric spatial structure on ULUE. Second, the second-order coefficient of polycentric spatial structure index was added to the model in order to observe whether the impact of polycentric spatial structure on ULUE was nonlinear. The intermediary effect model is employed to explore the impact mechanism of the polycentric spatial structure on ULUE. The moderating effect of the infrastructure level are also considered in this paper. Third, most studies ignore the endogenous relationship between the spatial agglomeration and ULUE. To overcome the endogeneity in the model, an urban primacy index to construct the instrumental variables for the polycentric index was used.

## 3. Mechanism Analysis and Research Hypothesis

**Figure 1:**
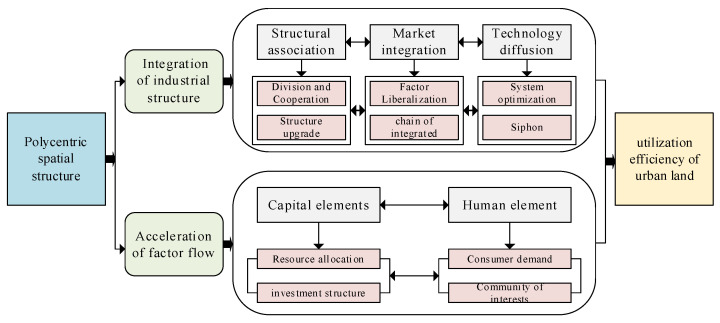
Diagram of the intermediary effect.

After sorting out the relevant literature, this paper believes that the impact of a polycentric spatial structure of UA on ULUE mainly lies in whether to improve its land use efficiency. With the implementation of the polycentric development strategy, the regional economic development pattern has gradually changed. The level of coordination within UAs is increasing and interaction is more frequent. A diagram of the influence mechanisms is shown in Figure 1.

### 3.1. The Polycentric Spatial Structure of UA Has a Phased Impact on ULUE

According to the theory of an agglomeration economy, the essence of a polycentric urban spatial structure is the relatively balanced distribution of various development factors among different cities in a region [50,51]. The reasonable urban spatial structure of UA is considered as an effective way to improve the ULUE. Although it is necessary to avoid the development model of “excessive monocentric spatial structure of UA”, the over-dispersed development model of UAs is likely to lead to a relative fragmentation of its industrial organization and labor distribution [52]. Competition among cities outweighs cooperation, and market integration and factor flow are restrained. Excessive single and dispersed spatial structures are not conducive to the improvement of urban land use efficiency. Judging from the development history of UAs, China’s urban system is not mature, and the radiation and driving effects of the central cities in UA have not yet fully played their role [53]. As Xia et al. [54] emphasized, in the long run, the improvement of regional production efficiency still needs to be driven by the central cities. However, when the “leading position” of central cities is too high, the marginal output of high-quality production factors is also limited. UA starts to develop towards the polycentric. Therefore, as China enters a stage of high-quality development, affected by the idea of “balanced regional development,” cities at all levels will have accelerated land supply and construction of new urban areas. However, a sprawling spatial structure reduces the economic agglomeration effect and resource efficiency, possibly reducing ULUE. Therefore, an appropriate polycentric spatial structure of UA is helpful to improve the level of ULUE, but an excessively high polycentric level may not be conducive to the improvement of ULUE.

**H1.**  *The influence of a polycentric spatial structure of UA on ULUE has an inverted U-shaped characteristic. The polycentric spatial structure of UA can promote ULUE to a certain extent. However, when the polycentricity is too high, the polycentric spatial development model is not conducive to the improvement of ULUE.*

### 3.2. Mechanism between Polycentric Spatial Structure and ULUE

#### 3.2.1. Improve the Integration Level of Industrial Structure

At present, polycentric development has become the typical implementation path of spatial planning of UAs in China [18]. This section mainly analyzes the path of industrial structure integration based on the framework of “structure-market-technology”.

First, structural association. With the gradual formation of the polycentric spatial pattern of the UA, a regional cooperation system of functional division of labor and market sharing has been formed spontaneously within the UA. Different cities can divide industries within the UA, and promote the overall upgrading of the region’s industrial structure [55]. In particular, the transformation and upgrading of an industrial structure can directly reduce the constraints of traditional extensive production factors on land economic output. It can also optimize the industrial spatial structure within a UA, promote the efficient circulation and intensive utilization of elements required for high-quality development and jointly improve the ULUE. Second, market integration. A regional polycentric spatial structure can promote the marketization of production factors and provide endogenous power for their efficient allocation. It makes the factor market within the UA continuously perfect and forms a relatively complete industrial chain in the region. UA takes the construction of a complete industrial chain to optimize the regional spatial layout. As a result, the efficiency of integrated urban land use has increased. Third, technology diffusion. The polycentric spatial structure of UA is conducive to the efficient integration of industrial technology. It also contributes to the overall optimization of regional innovation systems. Technological innovation in land use can achieve the effect of doubling the output value per unit of land. Thus, ULUE is improved.

#### 3.2.2. Speed Up the Mobility of Factors

This part refers to the research of Wan et al. [56,57] to investigate the driving mechanism of the factor flow mainly from the two main dimensions of capital factor mobility and labor factor mobility.

First, capital factor mobility. Polycentric spatial structure provides a good environment for the clustering and diffusion of capital elements among cities [13]. Polycentric spatial structure can change the resource distribution and investment structure of UA and improve the efficiency of capital factor allocation. It can also enhance the interaction between cities within a UA and promote the improvement of the integrated benefits of land use. Second, labor factor mobility. Labor is the most basic factor of production required for all productive activities. The agglomeration effect and scale effect produced by a labor factor flow can reduce the demand of economic development for land construction. It can decompose land production costs and promote the improvement of ULUE. In addition, in the process of factor mobility, regional interest communities can be formed [58]. People’s sense of regional identity is strengthened and the benefits of integrated urban land use are enhanced.

**H2:**  *The polycentric spatial structure of UA can affect ULUE in two ways: the integration of an industrial structure and the acceleration of factor mobility.*

## 4. Research Design and Index Selection

### 4.1. Study Area

**Figure 2:**
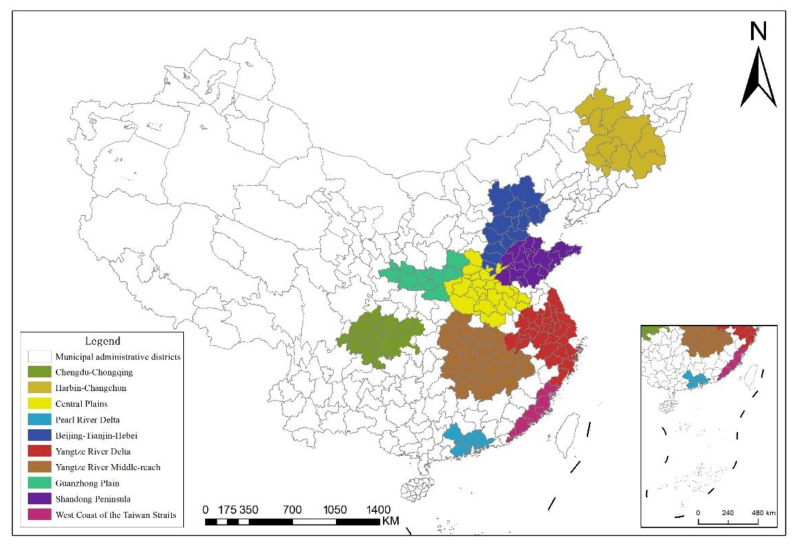
Location map of study area.

China has a large number of UAs with different dominant levels, and most of them are in the cultivation period. The agglomeration and radiation capacity of central cities is limited. Therefore, we selected ten UAs with relatively mature development in China as the research object. Excluding some cities with serious data loss, we covered a total of 161 Prefecture-Level cities, including the east, northeast, central and western regions on the spatial scale. The specific scope of the UA is determined according to the approval documents of the relevant UA planning. For example, the samples of the Yangtze River Middle-reach UA and Chengdu-Chongqing UA come from the Yangtze River Middle-reach UA Development Plan in April 2015 and the Chengdu-Chongqing UA Development Plan in March 2016, respectively. Considering the availability and integrity of data, 2004–2019 is selected as the time scale. The locations of the study area are demonstrated in Figure 2.

### 4.2. Data Sources

Combined with the availability of data, the time span selected in this paper covers the period from 2004–2019. The data originate from the China Urban Statistical Yearbook and China regional economic statistical yearbook. A few missing data were filled in by consulting the statistical yearbooks of each city or using the interpolation method.

### 4.3. Methodology

#### 4.3.1. Polycentric Spatial Structure

Urban spatial structure is the core variable tested in this paper. Referring to the methods of Meijers and Burger [59], this paper mainly uses the urban order size distribution rule to construct the polycentricity index of UA, and measures the polycentric spatial structure of UA from the perspective of morphology. The equation is as follows.

(1) $$\ln R_{it}^{\prime }=C-p\ln R_{it}$$

In the above equation, $R_{it}$ represents the city size of city i in the year t. Generally speaking, the city size is represented by the number of urban population or the level of economic development. As the focus of this study is the ULUE of UA, this paper uses the practices of Yan and Wang [8] for reference and selects the city GDP to measure the city size from an economic perspective. C is a constant, and $R_{it}^{\prime }$ represents the rank of the city size of the city i of UA in the year t. After ranking the city size of each city in each UA, the value is substituted into Equation (1) for regression to obtain the polycentric spatial structure index p of the UA in year t. Generally speaking, the greater the absolute value of p, the stronger the polycentricity of the region.

#### 4.3.2. ULUE of UA

At present, the super efficiency SBM model proposed by Tone [60] is often used to measure ULUE. As the super efficiency SBM model is the same as the traditional DEA model, when its decision-making unit is in a technically effective state, the comprehensive efficiency measurement value is 1, so it is difficult to measure the efficiency of multiple effective decision-making units [61,62], and when the evaluated decision-making unit data is panel data containing multiple time point observations, it is necessary to analyze the change of its production efficiency with a time series [63]. In order to solve this problem, the academic circles often use the combination of super efficiency SBM and the Malmquist index to measure the total factor productivity and study its influencing factors [61,64]. Based on the research of Chen et al. [65], Jin et al. [66] and Zhang et al. [67], this paper adopts the Malmquist index based on super efficiency SBM as the measurement method of ULUE of UA as the explained variable in the measurement model. This paper measures the ULUE of UA based on the MaxDEA 6.4 software. The input–output index system of ULUE is shown in Table 1 below.

#### 4.3.3. Benchmark Regression Model

The change of a single or polycentric spatial structure of UA takes a long time, so it can be considered that the polycentric index is exogenous to ULUE in a short time. Based on this, OLS estimation is used to analyze the impact of a polycentric spatial structure on ULUE. In order to capture the possible U-shaped or inverted U-shaped curve relationship between them, the quadratic term of a polycentric index is introduced to test this effect. As ULUE is also affected by economy, technology and systems, we also add the following control variables to the model:

Economic development level ($Eco_{it}$), measured directly by the GDP growth rate; the level of opening to the outside world ($Ope_{it}$), measured by the proportion of total foreign direct investment in GDP; the degree of government intervention ($Gov_{it}$), measured by the proportion of fiscal expenditure in GDP; human capital ($Huc_{it}$), expressed by the number of college students per 10,000 people; the per capita wage level ($Wag_{it}$) is represented by the average annual actual wage of urban workers. Finally, the benchmark model for investigating the impact of a polycentric spatial structure on ULUE is set as follows:

(2) $$L_{it}=\beta_{0}+\beta_{1}polycentric_{it}+\beta_{2}polycentric_{it}^{2}+\beta CV_{it}+\varepsilon_{it}$$

In the above equation, $CV_{it}$ includes the control variables in the regression model. $L_{it}$ is the ULUE, $polycentric_{it}$ is the polycentric spatial structure index, and $\beta_{0}\sim \beta_{8}$ is the constant term and the coefficients of each explanatory variable.

## 5. Empirical Test and Analysis

### 5.1. Polycentric Index and ULUE Measurement

The measurement results of polycentricity of China’s top ten UAs in some years are shown in Table 2 below, which can be roughly divided into three categories. The first category is the UA with the increase of polycentricity, including the Yangtze River Delta, Shandong Peninsula, Harbin-Changchun and the Pearl River delta UA. Its spatial structure evolves towards a multi-center, and the agglomeration degree of core cities shows a downward trend. The second category is the UA with a fluctuating spatial structure but is relatively stable, including Beijing-Tianjin-Hebei, the west coast of the Taiwan Straits, the Yangtze River Middle-reach and the Central Plains UA. There are many core cities and the level gap between cities is not obvious. The third category is the UAs with declining polycentricity, including Guanzhong Plain and Chengdu-Chongqing UAs, whose core cities are gradually prominent and the gap between urban levels widens. Among them, the first city in Guanzhong UA is larger, and the spatial structure of a single center is the most typical. From the perspective of regional location comparison, the UAs in the eastern region approximately obeys the Zipf’s law exponent [68]; the UAs in the central region have a weak polycentric structure and the UAs in the western region generally present a typical single-center structure.

Table 3 below shows the measurement results of ULUE of the top ten UAs in some years. On the whole, the total factor production index of ULUE of the top ten UAs in most years is greater than 1, indicating that their ULUE has been improved in most years. From the perspective of a time series, the change degree of the ULUE value of China’s top ten UAs from 2004 to 2019 is different, but the range is small. The average ULUE value gradually rises from 1.090 in 2005 to 1.133 in 2019. From the perspective of spatial distribution, the ULUE values of different regions are quite different. The ULUE values of UAs in eastern China are relatively high, whereas those in central and western China are relatively low. In 2004, the ULUE of Yangtze River Delta and Shandong Peninsula UAs were the highest, at 1.118 and 1.079, respectively. Second, the Beijing-Tianjin-Hebei and Pearl River Delta UAs were 1.075 and 1.068, respectively. However, the UAs in Guanzhong Plain and Chengdu-Chongqing were relatively low, with ULUE values of 1.003 and 1.002. By 2019, the change range of urban agglomerations will be roughly the same. The ULUE of UAs in the Yangtze River Delta and Shandong Peninsula will still be the highest, whereas that in the central and western regions will be lower.

### 5.2. Regression Results and Analysis

#### 5.2.1. Basic Regression Analysis

Firstly, this paper carries out benchmark regression on the econometric model of Equation (2). The results are shown in Models 1–6 in Table 4. Equations (1)–(6) give the results of the OLS estimator, fixed effect estimator and random effect estimator, respectively. Since the Hausman specification test rejected the random effect specification (Chis-q = 20.83, *p* value = 0.000), the results showed that the fixed effect regression was better. Through an LR test, this paper finally selects the time individual double fixed effect regression model, namely Model (4). It can be found that the estimation coefficients of the primary term of the polycentricity index are significantly positive, and the estimation coefficients of the corresponding square term are significantly negative, whether the control variable is added or not. This means that the impact of polycentric spatial structure on the ULUE of UA shows an obvious inverted U-shaped feature. Hypothesis 1 has been verified.

This phenomenon can be understood as that in the early stage of the formation of a polycentric spatial structure of UA, the polycentricity of a regional spatial structure is low. There is a significant development gap between central and surrounding cities. In the relatively low levels of polycentric spatial structure development, the effect of “scale borrowing” between surrounding cities and core cities is more significant. Thus, the ULUE in the region can be improved with the development of a polycentric spatial structure. However, China’s UAs are still in the development stage, and the regional central cities have not yet reached the optimal scale. Over-equilibrium spatial structures within regions reduce the economic agglomeration effects. This is detrimental to the radiation and driving role of the central city within the region. In addition, industry is still the main driving force of China’s urban economic development at this stage. Too flat of a polycentric spatial structure can easily lead to industrial dispersion, repeated construction and the phenomenon that cooperation between cities is greater than the competition. This would reduce the mobility of production factors and the degree of market integration. Therefore, an excessively high level of a polycentric spatial structure reduces the ULUE of UA.

#### 5.2.2. Robustness Test

The results in Table 4 provide the preliminary proof for us to understand the impact of a polycentric spatial structure on the ULUE of UA. However, in view of the possible errors and endogenous problems in the process of model setting, a robustness test is conducted on the basis of the above research. First, this paper brings the explanatory variable into the model for verification, aiming to weaken the endogenous issue that may be caused by two-way causality; secondly, this paper uses the city primacy ($monopro_{it}$) as a substitute index for the polycentricity index to carry out a regression estimation again. The $monopro_{it}$ index is calculated by the proportion of the population size of the first city in the population size of the whole urban agglomeration. Table 5 reports the regression results for Equation (3). In Table 5, columns (1–2) report the result of changing the core explanatory variable to urban primacy. Columns (3–4) are the result of delaying the polycentric index by one period, and columns (5–6) are the result of adding the interactive term between the polycentric index and ULUE. The above regression results show that the greater the primacy of the central city, the urban agglomeration develops to a single center, which has a negative effect on the ULUE of the UA. The results of one lag period are consistent with the previous test results, and the addition of interactive items significantly indicates that there is a regulatory effect. The above results further verify the robustness of this conclusion.

## 6. Transmission Mechanism Test

### 6.1. Intermediary Effect Estimation

After proving the impact of a polycentric spatial structure on ULUE, combined with the previous theoretical mechanism analysis, this paper uses the mediating effect model to reveal the mechanism. The mediating effect model is shown in Equations (3)–(5).

(3) $$L_{it}=\beta_{0}+\beta_{1}polycentric_{it-1}+\beta_{2}polycentric_{it-1}^{2}+\beta CV_{it-1}+\varepsilon_{it-1}$$

(4) $$M_{it}=\eta_{0}+\eta_{1}polycentric_{it-1}+\eta_{2}polycentric_{it-1}^{2}+\eta CV_{it-1}+\varepsilon_{it-1}$$

(5) $$L_{it}=\gamma_{0}+\gamma_{1}polycentric_{it-1}+\gamma_{2}polycentric_{it-1}^{2}+\gamma_{3}M_{it}+\gamma CV_{it-1}+\varepsilon_{it-1}$$

In the above equations, $M_{it}$ are intermediary variables, including industrial structure integration ($Isr_{i}$) and factor flow ($Flow_{i}$). Industrial structure integration variable, which reflects the dual changes of the proportional relationship of each industry in “quantity” and the labor productivity in “quality”. Referring to the practices of Yu and Su [69], this paper defines the level of industrial structure integration as the weighted value of the product of the proportional relationship between industries and labor productivity (Equation (6)); the factor flow is reflected by the ratio of urban passenger traffic volume to highway mileage of UAs based on the practice of Liu et al. [70]. The remaining variables are the same as above.

(6) $$Isr_{i}=\sum_{m=1}^{3}\frac{Y_{imt}}{Y_{it}}\times \ln\left(\frac{P_{imt}}{P_{mt}}\right)=\sum_{m=1}^{3}\frac{Y_{imt}}{Y_{it}}\times \ln\left(\frac{Y_{imt}}{L_{imt}}/\frac{Y_{mt}}{L_{mt}}\right),\;m=1,2,3$$

In the above equation, $Y_{imt}$ represents the added value of the gross domestic product of the m industry of the city i in the period of t; $L_{imt}$ refers to the number of employees in the m industry of the city i during t period; $P_{imt}$ refers to the labor productivity of the m industry of the city i in the period of t. In order to ensure the stability of the data, the two intermediary variables are treated logarithmically in this paper. Considering the lag effect of the explanatory variables on the intermediary variables and dependent variables, the lag of the explanatory variables is substituted into the regression Equations (3)–(5).

The specific empirical test is divided into three steps: first, regression is carried out for Equation (3). If the coefficients $\beta_{1}$ and $\beta_{2}$ are significant, it indicates that the impact of the polycentric spatial structure on ULUE is significant without intermediary variables; Then, the Equation (4) is regressed to investigate whether there is an inverted U-shaped relationship between the polycentric spatial structure index and the intermediary variables, with coefficients $\eta_{1}$ and $\eta_{2}$. Then, the intermediary variables and polycentric spatial structure index are introduced into Equation (3) to form Equation (5), and the direct effects $\gamma_{1}$, $\gamma_{2}$ and mediating effect $\gamma_{3}$ of polycentric spatial structure on ULUE are judged.

In Table 6, columns (1–2) show the estimation results with industrial structure integration as the explained variable. Columns (3–4) show the estimation results with factor flow as the explained variable. It can be found that the estimation coefficients of the first-order coefficients of the polycentric spatial structure index are significantly positive, whereas the estimation coefficients of the second-order coefficients are significantly negative, and the significance level is more than 5%. This means that with the improvement of the polycentric spatial structure level of UA, the integration degree of industrial structure and the level of factor mobility also show an inverted U-shaped change trend. This change feature corresponds to the relationship between the polycentric spatial structure and ULUE. It also explains the reason the polycentric spatial structure of UA can have an inverted U-shaped impact on ULUE. To sum up, we can also preliminarily determine that the integration of industrial structure and factor flow are important intermediary factors for the polycentric spatial structure of UA to affect ULUE.

Next, this paper further uses the mediating effect model to test the mechanism of polycentric spatial development model affecting ULUE. Based on the methods of Chen and Qiu [71], the fitting values of the two mediators of integration of industrial structure and factor flow are calculated according to the results of Columns (1) and (3) in Table 6. Then, we take the ULUE as the explained variable, and take the fitting values of the two intermediary variables as explanatory variables into Equation (5) for regression estimation. The estimation results are shown in Table 7. In the regression results without considering the polycentric spatial structure, the estimated coefficients of the fitting variables of market integration and factor mobility are positive and significant at the significance level of 5%. After adding the index of polycentricity, it can be found that the integration of industrial structure and factor mobility have all passed the significance test. This shows that the integration of industrial structure and factor flow play an important mediating effect in the process of a polycentric spatial structure affecting ULUE, but it is not a complete mediating effect. There are still other intermediary factors that are not captured in this paper. At the same time, Hypothesis 2 has been verified.

### 6.2. Moderating Effect Estimation

Perfect Base Facilities can promote the interaction among people, things and information in UAs. The development level of infrastructure should also include the effect of a polycentric spatial structure on ULUE. Therefore, this paper adds the interaction term $polycentric_{it-1}\times Inf_{it-1}$ and its square term between the index of polycentricity and infrastructure to the benchmark regression model (3). We use this method to examine the moderating effect of infrastructure level. Referring to the practice of Liu and Hu [72], this paper uses the highway mileage of each city and divides it by the area of the city to obtain the level of transportation infrastructure. We also use the per capita post and telecommunications business volume of each city to represent the level of communication infrastructure. In order to ensure the stability of the data, this paper deals with the logarithm of the two moderators.

Table 8 shows the moderating effect test results. It can be found that the estimation coefficients of the interaction term between the polycentric spatial structure index and the infrastructure first-order coefficients are negative, but the interaction term with its second-order coefficients are positive. This shows that level of infrastructure has played an obvious positive U-shaped moderating effect in the process of polycentric spatial structure affecting ULUE. This can be explained as follows: under the condition of perfect infrastructure, the polycentric spatial development model of a UA can more effectively promote the improvement of ULUE. This is also consistent with practical experience. In UAs with lagging transportation and communication infrastructure, even if the urban scale system presents a polycentric spatial pattern, it will be difficult to realize the optimal allocation of production factors due to the high communication costs between cities. In addition, the polycentric spatial structure index of a UA and the estimated coefficient of communication infrastructure are not significant. The reason may be that, because of regional differences, the development speed and level of communication infrastructure in the eastern region and the central and western regions are different. The coefficient is not significant because of regional heterogeneity, but it is still positively U-shaped as a whole.

## 7. Discussion

In this paper, we show that the influence of a polycentric spatial structure of UA on ULUE shows significant inverted U-shape characteristics. The integration of industrial structure and factor mobility play a significant intermediary role. The infrastructure level plays a positive U-shaped regulatory role. The empirical results of this research add to the growing literature concerning the effectiveness of polycentric development in improving resource utilization efficiency and promoting environmental sustainability.

The above analysis shows that the polycentric spatial structure of a UA could effectively improve ULUE. This finding is similar to the research results of Yan and Wang [8]. We consider whether this result means that the continuous development of the polycentric spatial planning is the future development direction of all UAs. In fact, this paper proves that the influence of a polycentric spatial structure of UA on ULUE is nonlinear. Too high or too low a polycentric spatial structure of UAs is not conducive to the improvement of ULUE. This finding is similar to that of Chen et al.’s [42]. However, what they explored was the impact of a polycentric spatial structure on energy efficiency. However, the research results of this paper are different from those of Han and Chen [73], who believe that the higher the polycentric spatial structure is, the better the industrial enterprises’ emissions will be. In addition, the existing research neglects the endogenous relationship between spatial agglomeration and ULUE [8]. In order to overcome the endogeneity in the model, this paper uses an urban primacy index to construct the instrumental variables for the polycentric index. The influence of the polycentric spatial structure of UA on ULUE presents a significant inverted U-shaped feature. An appropriate polycentric development strategy can be an effective tool to promote the efficiency of land resource utilization.

Some scholars have found that a polycentric spatial structure will promote the improvement of energy utilization efficiency [74], economic efficiency [75], green land use efficiency [8], etc. Although there are important discoveries revealed by these studies between polycentric spatial structures and efficiency, there are also limitations. They did not delve into the impact mechanism between the two. This paper can make a powerful supplement for the research in this area. Here, we describe two channels where polycentric spatial structure affects UEUL. This section is also one of the highlights of this paper. First, the polycentric spatial structure enhances the integration level of an industrial structure of UA. This result is consistent with Lu et al. [4] and Li et al. [40]. In the polycentric spatial pattern, the cooperation system of functional division and market sharing has spontaneously formed within the UA, forming a relatively complete industrial chain. The overall innovation system of the UA has been continuously optimized, and the sharing and utilization rate of public resources have been constantly improved [76]. This can directly reduce the restriction of traditional extensive production factors on the improvement of land economic output, achieve the effect of doubling the production value of unit land and then improve the efficiency of urban land use. Second, the polycentric spatial structure of UA accelerates the mobility of factors in UA. This result is consistent with Lu et al. [4] and Odell et al. [77]. The polycentric spatial structure of UA can provide a good environment for the concentration and diffusion of capital elements among cities, and improve the efficiency of resource allocation of UA. The level of infrastructure has played an obvious positive U-shaped moderating effect in the process of polycentric spatial structure affecting ULUE. Specifically, we divide the infrastructure into transportation infrastructure and communication infrastructure. The impact of transportation infrastructure on ULUE is significant, but the impact coefficient of information infrastructure is not significant. The research by Lu et al. [78] have come to similar conclusions but they did not consider the impact of communication infrastructure. From the perspective of improving the UEUL, regions with better transport infrastructure are more suitable for developing a polycentric spatial structure. Our research enriches the scope of research on the impact of infrastructure on urban land use systems.

However, this study still has some limitations. First, because of the availability of data, this analysis of the impact of polycentric spatial structure on ULUE only stays in the top ten UAs in China. Whether the survey results are also applicable to other urban agglomerations in the world needs to be analyzed separately. Second, this research employs socio-economic data to characterize spatial structure. Other disciplines also have different methods and different types of data to explore spatial structure. However, our results provide decision-making materials for sustainable use of land resources and high-quality economic development in China.

## 8. Conclusions

On the basis that the close relationship between regional spatial structure and ULUE has been confirmed, this study uses the data of China’s top ten UAs from 2004 to 2019 to further explore the dynamic impact and mechanism of a polycentric spatial structure on ULUE. The specific research conclusions of this paper are as follows:

(1) The polycentric spatial structure of UAs in the eastern region approximately obeys the Zipf’s law exponent [68], and the UAs in the central and western regions show a weak polycentric structure, but the polycentric degree shows different characteristics from 2004 to 2019. The ULUE values of the ten UAs are quite different. The eastern UAs are relatively high, and the overall change range is small, but the overall trend is upward. (2) After controlling the UAs’ scale, human capital, government intervention and other factors, a polycentric spatial structure of UA positively affects ULUE. An inverted U-shaped curve describes the relationship between a polycentric spatial structure and ULUE after adding the quadratic term. Although a polycentric spatial structure’s effect on ULUE is positive overall, the impact mechanism may change this effect from positive to negative. (3) Regarding the aspect of mechanism, the integration of industrial structure and factor mobility are important ways for the polycentric spatial structure of UA to affect ULUE. The mediating variable of integration of industrial structure and factor mobility has a positive and partially mediating effect between a polycentric spatial structure and ULUE. In the process of a polycentric spatial structure affecting ULUE, infrastructure level plays a positive U-shaped regulatory role. Based on the above research conclusions, the following policy recommendations are made.

(1) UAs should implement a polycentric development strategy to relieve the non-core functions of central cities. In UA planning, we should give full play to the positive role of a polycentric spatial structure in improving land use efficiency. However, it is also necessary to clarify the centrality of central cities in the region and to avoid over-flattening development patterns. The government promotes the gradient development of the surrounding cities by consolidating the dominance of the central cities of the region and strengthening their diffusion effects. By speeding up the construction of a polycentric system of UAs, the distribution of population and economic activities in a central city is avoided. (2) In the process of promoting the polycentric layout of UAs, close cooperation between cities within UAs needs to be strengthened. In addition, another external condition that a polycentric spatial structure can improve ULUE is the free flow of elements. In the context of local protection and market fragmentation in China, we need to improve communication mechanisms between regional governments. We can build industrial parks and unified factor markets to minimize administrative barriers and market boundaries. The government should promote the construction of transport and communication infrastructure in urban agglomerations. By strengthening the spatial linkages between cities, the negative impact of geographical distance on the development of polycentric spatial structure can be weakened. (3) We need to actively promote the efficient integration of intercity cultures within urban agglomerations. This would increase the willingness to cooperate between cities and increase the sense of regional identity. Through strengthening human capital, we can strengthen the agglomeration capacity and radiation effect of the whole region. All parties should work together to promote the high-quality economic development of UA and the efficient and rational use of land.

## Figures and Tables

**Table 1:** Input–output index system of ULUE.

Indicator Type	Definition of Indicators	Index Composition	Unit
Input indicators	Land	Urban construction land area	km^2^
	Capital	Social fixed assets investment	Ten thousand yuan
	Labor force	Employment number of secondary and tertiary industries	Ten thousand people
Output indicators	Economic benefits	Added value of secondary and tertiary industries	Ten thousand yuan
	Environmental benefits	Green area	km^2^
	Societal benefits	General budgetary revenue	Ten thousand yuan

**Table 2:** Polycentricity of top ten UAs in particular years.

Urban Agglomeration	2004	2008	2012	2016	2019
Yangtze River Delta	0.763	0.796	0.842	0.845	0.849
Shandong Peninsula	1.394	1.515	1.700	1.689	1.791
Chengdu-Chongqing	0.904	0.744	0.847	0.685	0.788
Guanzhong Plain	0.772	0.756	0.795	0.779	0.738
Harbin-Changchun	0.752	0.869	0.951	1.025	0.816
West Coast of the Taiwan Straits	0.920	1.011	0.992	0.993	0.866
Beijing-Tianjin-Hebei	0.974	0.894	0.846	0.823	0.828
Yangtze River Middle-reach	1.222	1.290	1.260	1.228	1.179
Central Plains	1.516	1.510	1.557	1.530	1.486
Pearl River Delta	0.908	0.742	0.787	0.795	0.748

**Table 3:** ULUE values of top ten urban agglomerations in specific years.

Urban Agglomeration	2004	2008	2012	2016	2019
Yangtze River Delta	1.180	1.278	1.298	1.338	1.677
Shandong Peninsula	1.079	1.150	1.108	0.912	1.505
Chengdu-Chongqing	1.003	1.208	1.064	0.924	1.026
Guanzhong Plain	1.002	0.978	1.179	1.056	1.015
Harbin-Changchun	1.062	1.220	1.432	1.510	0.988
West Coast of the Taiwan Straits	1.017	1.064	1.098	0.940	1.001
Beijing-Tianjin-Hebei	1.075	1.076	1.059	1.024	1.036
Yangtze River Middle-reach	1.033	1.166	1.358	0.996	0.976
Central Plains	0.988	0.884	0.922	1.063	1.018
Pearl River Delta	1.068	1.021	0.969	1.042	0.929

**Table 4:** Impact of polycentric spatial structure on ULUE (Full sample test).

Variable	POOL (1)	POOL (2)	FE (3)	FE (4)	RE (5)	RE (6)
$polycentric_{it}$	1.204 * (1.77)	1.004 * (1.42)	4.202 *** (3.56)	4.503 *** (3.65)	1.469 * (1.84)	1.582 * (1.84)
$polycentric_{it}^{2}$	−0.518 * (−1.80)	−0.475 * (−1.60)	−1.258 *** (−2.65)	−1.393 *** (−2.80)	−0.616 * (−1.82)	−0.674 * (−1.82)
Constant term	0.427 (1.15)	0.892 * (1.97)	−1.800 ** (−2.52)	−1.711 ** (−2.18)	0.245 (0.55)	0.272 (0.49)
Control variable	NO	YES	NO	YES	NO	YES
Individual fixation	NO	NO	YES	YES	YES	YES
Time fixation	NO	NO	YES	YES	YES	YES
Adj R^2^	0.009	0.003	0.228	0.240	0.147	0.155

Notes: (1) Standard errors are shown in parentheses below parameter estimates. (2) ***, ** and * denote significance at the 1%, 5% and 10% level, respectively.

**Table 5:** Regression result analysis of robustness test.

Variable	(1)	(2)	(3)	(4)	(5)	(6)
$monopro_{it}$	−2.169 * (−1.87)	−2.522 * (−1.72)				
$polycentric_{it-1}$			2.769 ** (2.40)	3.071 *** (2.58)		
$polycentric_{it-1}^{2}$			−0.651 * (−1.37)	−0.831 * (−1.65)		
$polycentric_{it}*L_{it}$					0.918 *** (37.82)	0.919 *** (37.88)
Constant term	1.605 *** (5.65)	1.544 *** (4.26)	−1.041 (−1.51)	−0.689 * (−0.95)	0.073 *** (2.12)	−0.037 (−0.43)
Control variable	NO	YES	NO	YES	NO	YES
Individual fixation	YES	YES	YES	YES	YES	YES
Time fixation	YES	YES	YES	YES	YES	YES
Adj R^2^	0.134	0.137	0.211	0.2313	0.3343	0.3491

Note: * represents *p* < 10%, ** represents *p* < 5%, *** represents *p* < 1%, respectively. Value in parentheses is the *t*-test value.

**Table 6:** Mechanism test of polycentric structure affecting ULUE.

Variable	Industrial Structure Integration (1)	Industrial Structure Integration (2)	Factor Flow (3)	Factor Flow (4)
$polycentric_{it-1}$	3.451 *** (2.73)	3.867 ** (2.28)	4.714 *** (3.52)	4.683 *** (3.86)
$polycentric_{it-1}^{2}$	−1.872 *** (−3.58)	−1.992 *** (−2.80)	−2.349 *** (−4.24)	−2.508 *** (−4.89)
$Eco_{it-1}$		1.224 ** (2.07)		−1.478 *** (−2.64)
$Ope_{it-1}$		−8.581 ** (−2.20)		−7.814 ** (−2.45)
$Gov_{it-1}$		0.982 (0.55)		5.231 ** (2.09)
$Huc_{it-1}$		0.007 *** (5.22)		0.004 *** (2.98)
$Wag_{it-1}$		−0.001 *** (−8.95)		−0.001 *** (−5.51)
Individual fixation	YES	YES	YES	YES
Time fixation	YES	YES	YES	YES
Constant term	−1.505 ** (−1.98)	−2.626 *** (−2.54)	−2.025 ** (−2.52)	−2.234 *** (−3.02)
Adj R^2^	0.727	0.816	0.771	0.829

Note: ** represents *p* < 5%, *** represents *p* < 1%, respectively. Value in parentheses is the *t*-test value.

**Table 7:** Test of mediating effect of polycentric spatial structure on ULUE.

Variable	UEUL (1)		UEUL (2)	
$polycentric_{it-1}$		2.481 ** (2.09)		3.051 ** (3.55)
$polycentric_{it-1}^{2}$		−0.657 ** (−1.08)		−1.231 ** (2.12)
$Inst_{i}$	1.236 ** (1.13)	1.621 ** (1.39)		
$Flow_{i}$			2.011 ** (2.13)	2.321 ** (2.68)
Control variable	YES	YES	YES	YES
Individual fixation	YES	YES	YES	YES
Time fixation	YES	YES	YES	YES

Note: ** represents *p* < 5%. Value in parentheses is the *t*-test value.

**Table 8:** Test the moderating effect of infrastructure.

Variable	Transportation Infrastructure		Communication Infrastructure	
$polycentric_{it-1}$	3.949 *** (3.05)	4.126 *** (3.10)	2.907 *** (2.50)	3.347 *** (2.75)
$polycentric_{it-1}^{2}$	−1.136 ** (−2.00)	−1.213 ** (−2.09)	−0.716 * (−1.49)	−0.965 * (−1.87)
$polycentric_{it-1}\times Inf_{it-1}$	−0.371 * (−1.74)	−0.431 * (−1.77)	−0.687 (−1.33)	−0.793 (−1.19)
$polycentric_{it-1}\times {Inf_{it-1}}^{2}$	0.214 ** (2.10)	0.204 * (1.80)	0.043 (1.23)	0.048 (1.809)
Constant term	−2.12 *** (−2.63)	−2.626 *** (−2.54)	1.55 (0.77)	2.308 (0.91)
Control variable	NO	YES	NO	YES
Individual fixation	YES	YES	YES	YES
Time fixation	YES	YES	YES	YES
Adj R^2^	0.2498	0.816	0.771	0.829

Note: * represents *p* < 10%, ** represents *p* < 5%, *** represents *p* < 1%, respectively. Value in parentheses is the *t*-test value.

## Data Availability

The data presented in this study are available on request from the corresponding author. The data are not publicly available due to privacy.

## References

[1] Yang K., Zhong T., Zhang Y., Wen Q. (2020). Total factor productivity of urban land use in China. Growth Chang..

[2] Lin Q., Ling H. (2021). Study on Green Utilization Efficiency of Urban Land in Yangtze River Delta. Sustainability.

[3] Zhang H., Chen M., Liang C. (2022). Urbanization of county in China: Spatial patterns and influencing factors. J. Geogr. Sci..

[4] Lu F., Liu M., Wang W., Liu M. (2022). Impact of Polycentric Urban Network on Industrial Structure Upgrades: Evidence from the Yangtze River Economic Belt. J. Urban Plan. Dev..

[5] Luo X., Tong Z., Xie Y., An R., Yang Z., Liu Y. (2022). Land Use Change under Population Migration and Its Implications for Human-Land Relationship. Land.

[6] Zhang P., Zhao Y., Zhu X., Cai Z., Xu J., Shi S. (2020). Spatial structure of urban agglomeration under the impact of high-speed railway construction: Based on the social network analysis. Sustain. Cities Soc..

[7] Zhou N. (2022). Research on urban spatial structure based on the dual constraints of geographic environment and POI big data. J. King Saud Univ. Sci..

[8] Yan S., Wang J. (2022). Does Polycentric Development Improve Green Utilization Efficiency of Urban Land? An Empirical Study Based on Panel Threshold Model Approach. Land.

[9] Meijers E.J., Burger M.J., Hoogerbrugge M.M. (2016). Borrowing size in networks of cities: City size, network connectivity and metropolitan functions in Europe. Pap. Reg. Sci..

[10] Yu X., Liu Y., Zhang Z., Xiong Y., Dang M. (2022). Urban spatial structure features in Qinling mountain area based on ecological network analysis-case study of Shangluo City. Alex. Eng. J..

[11] Ouwehand W.M., van Oort F.G., Cortinovis N. (2022). Spatial structure and productivity in European regions. Reg. Stud..

[12] Chen D., Lu X., Zhang C. (2021). Study on the direct-driving and spatial-spillover effects of regional integration on urban land use efficiency. Inq. Into Econ. Issues.

[13] Song J., Chen S. (2021). Impact of economic agglomeration on land use eco-efficiency of three major urban agglomerations in China. J. Nat. Resour..

[14] Yu B., Su Y. (2020). Research on the impact of industrial structure adjustment on land use efficiency and spillover effect: An empirical analysis based on PSDM model and PTR model. China Land Sci..

[15] Zhang W., Zou J. (2020). Effect of production factors on urban land use efficiency: Based on the provincial data of different development stages. Resour. Sci..

[16] Parr J.B. (2004). The polycentric urban region: A closer inspection. Reg. Stud..

[17] Veneri P., Burgalassi D. (2012). Questioning polycentric development and its effects. Issues of definition and measurement for the Italian NUTS-2 regions. Eur. Plan. Stud..

[18] Lanfredi M., Egidi G., Bianchini L., Salvati L. (2022). One size does not fit all: A tale of polycentric development and land degradation in Italy. Ecol. Econ..

[19] Li W., Sun B., Zhang T. (2019). Spatial structure and labour productivity: Evidence from prefectures in China. Urban Stud..

[20] Yue W., Wang T., Liu Y., Zhang Q., Ye X. (2019). Mismatch of morphological and functional polycentricity in Chinese cities: An evidence from land development and functional linkage. Land Use Policy.

[21] Liu X., Derudder B., Wang M. (2018). Polycentric urban development in China: A multi-scale analysis. Environ. Plan. B-Urban..

[22] Fei R., Lin Z., Chunga J. (2021). How land transfer affects agricultural land use efficiency: Evidence from China’s agricultural sector. Land Use Policy.

[23] Huang J., Xue D. (2019). Study on Temporal and Spatial Variation Characteristics and Influencing Factors of Land Use Efficiency in Xi’an, China. Sustainability.

[24] Ma J., Li W., Wang Z., He L., Han L. (2022). Measuring Multi-Faceted Land Use Efficiency of Large-Scale Urban Agglomerations under Multi-Scale Drivers: Evidence from China. Land.

[25] Zhang R., Lu J. (2022). Spatial-Temporal Pattern and Convergence Characteristics of Provincial Urban Land Use Efficiency under Environmental Constraints in China. Int. J. Environ. Res. Public Health.

[26] Zhao Q., Bao H.X.H., Zhang Z. (2021). Off-farm employment and agricultural land use efficiency in China. Land Use Policy.

[27] He Y., Xie H., Fan Y., Wang W., Xie X. (2016). Forested Land Use Efficiency in China: Spatiotemporal Patterns and Influencing Factors from 1999 to 2010. Sustainability.

[28] Xie H., Chen Q., Wang W., He Y. (2018). Analyzing the green efficiency of arable land use in China. Technol. Forecast. Soc. Chang..

[29] Hu H., Pan L., Jing X., Li G., Zhuo Y., Xu Z., Chen Y., Wang X. (2022). The Spatiotemporal Non-Stationary Effect of Industrial Agglomeration on Urban Land Use Efficiency: A Case Study of Yangtze River Delta, China. Land.

[30] Zhu X., Li Y., Zhang P., Wei Y., Zheng X., Xie L. (2019). Temporal-spatial characteristics of urban land use efficiency of China’s 35mega cities based on DEA: Decomposing technology and scale efficiency. Land Use Policy.

[31] Kuang B., Lu X., Zhou M., Chen D. (2020). Provincial cultivated land use efficiency in China: Empirical analysis based on the SBM-DEA model with carbon emissions considered. Technol. Forecast. Soc. Chang..

[32] Wang W., Dong F.I. (2020). Research on Land Use Efficiency of Zhongyuan Urban Agglomeration, International Workshop on Green Energy, Environment and Sustainable Development (G2ESD).

[33] Liu Z., Zhang L., Rommel J., Feng S. (2020). Do land markets improve land-use efficiency? evidence from Jiangsu, China. Appl. Econ..

[34] Wang S., Cebula R.J., Liu X., Foley M. (2021). Housing prices and urban land use efficiency. Appl. Econ. Lett..

[35] Wang P., Shao Z., Wang J., Wu Q. (2021). The impact of land finance on urban land use efficiency: A panel threshold model for Chinese provinces. Growth Chang..

[36] Yu J., Zhou K., Yang S. (2019). Land use efficiency and influencing factors of urban agglomerations in China. Land Use Policy.

[37] Yu Y., Luo N. (2022). Effect of land price distortion on land use efficiency: Evidence from China. Econ. Res.-Ekon. Istraz..

[38] Xie X., Fang B., Xu H., He S., Li X. (2021). Study on the coordinated relationship between Urban Land use efficiency and ecosystem health in China. Land Use Policy.

[39] Koroso N.H., Lengoiboni M., Zevenbergen J.A. (2021). Urbanization and urban land use efficiency: Evidence from regional and Addis Ababa satellite cities, Ethiopia. Habitat Int..

[40] Li W., Sun B., Zhang T., Zhang Z. (2022). Polycentric spatial structure and its economic performance: Evidence from meta-analysis. Reg. Stud..

[41] Yu H., Yang J., Li T., Jin Y., Sun D. (2022). Morphological and functional polycentric structure assessment of megacity: An integrated approach with spatial distribution and interaction. Sustain. Cities Soc..

[42] Chen X., Qiu B., Sun S. (2021). Polycentric spatial structure and energy efficiency: Evidence from China’s provincial panel data. Energy Policy.

[43] Huang Y., Liao R. (2021). Polycentric or monocentric, which kind of spatial structure is better for promoting the green economy? Evidence from Chinese urban agglomerations. Environ. Sci. Pollut. Res..

[44] Zhu K., Tu M., Li Y. (2022). Did Polycentric and Compact Structure Reduce Carbon Emissions? A Spatial Panel Data Analysis of 286 Chinese Cities from 2002 to 2019. Land.

[45] Ding C., Zhao X. (2014). Land market, land development and urban spatial structure in Beijing. Land Use Policy.

[46] Dominguez A., Enriquez Sierra H., Cuervo Ballesteros N. (2021). Regional Spatial Structure and Land Use: Evidence from Bogota and 17 Municipalities. Land.

[47] Li Z., Gurgel H., Li M., Dessay N., Gong P. (2022). Urban Land Expansion from Scratch to Urban Agglomeration in the Federal District of Brazil in the Past 60 Years. Int. J. Environ. Res. Public Health.

[48] Gao X., Zhang A., Sun Z. (2020). How regional economic integration influence on urban land use efficiency? A case study of Wuhan metropolitan area, China. Land Use Policy.

[49] Zhou Z., Li M. (2017). Spatial-temporal change in urban agricultural land use efficiency from the perspective of agricultural multi-functionality: A case study of the Xi’an metropolitan zone. J. Geogr. Sci..

[50] Tan X., Huang B., Batty M., Li J. (2021). Urban spatial organization, multifractals, and evolutionary patterns in large cities. Ann. Assoc. Am. Geogr..

[51] Liu P., Zhong F., Yang C., Jiang D., Luo X., Song X., Guo J. (2022). Influence mechanism of urban polycentric spatial structure on PM2.5 emissions in the Yangtze River Economic Belt, China. J. Clean. Prod..

[52] Meijers E., Hoogerbrugge M., Cardoso R. (2018). Beyond Polycentricity: Does Stronger Integration Between Cities in Polycentric Urban Regions Improve Performance?. Tijdschr. Econ. Soc. Ge..

[53] Pan S., Zhu D., Xu K. (2018). Are Chinese Cities Too Big or Too Small?—A Perspective of Labor Allocation Eficiency. Econ. Res. J..

[54] Xia C., Zhang A., Wang H., Liu J. (2020). Delineating early warning zones in rapidly growing metropolitan areas by integrating a multiscale urban growth model with biogeography-based optimization. Land Use Policy.

[55] Liu Y., Li S., Qin M. (2017). Urban spatial structure and regional economic efficiency: On the mode choice of urbanization development in China. Manag. World.

[56] Glaeser E.L., Ponzetto G.A.M., Zou Y. (2016). Urban networks: Connecting markets, people, and ideas. Pap. Reg. Sci..

[57] Wan Q., Yang L. (2021). Innovation factor flow and innovation ability of high-tech industries. Sci. Res. Manag..

[58] Tumwebaze H.K., Ijjo A.T. (2015). Regional Economic Integration and Economic Growth in the COMESA Region, 1980-2010. Afr. Dev. Rev..

[59] Meijers E.J., Burger M.J. (2010). Spatial structure and productivity in US metropolitan areas. Environ. Plan. A.

[60] Tone K. (2002). A slacks-based measure of super-efficiency in data envelopment analysis. Eur. J. Oper. Res..

[61] Lai Y., Xie J., Ye L., Ma X. (2021). Research on the efficiency and influencing factors of science and technology innovation in China: Based on the Super-Efficiency SBM-Malmquist-Tobit Model. Sci. Technol. Prog. Policy.

[62] Wang Y., Mao L., Wang Q. (2021). Research on the impact of environmental protection tax law on the innovation of Bejing-Tianjin-Hebei listed companies—Based on the analysis DEA-Malmquist index decomposition. China Soft Sci..

[63] You D., Di Y., Jiang K. (2017). The empirical study of Chinese regional scientific and technological innovation resources allocation efficiency—Based on output-oriented SBM and Malmquist Productivity Index. Soft Sci..

[64] Xie H., Wang W. (2015). Spatiotemporal differences and convergence of urban industrial land use efficiency for China’s major economic zones. J. Geogr. Sci..

[65] Chen W., Si W., Chen Z.-M. (2020). How technological innovations affect urban eco-efficiency in China: A prefecture-level panel data analysis. J. Clean. Prod..

[66] Jin L., Zhou T., Li J., Guo C., Wang J. (2021). Can niche suitability affect the innovation efficiency of public R&D organizations?—Empirical analysis based on regional comparison. China Soft Sci..

[67] Zhang K., Zhang J. (2022). Polycentricity and green development efficiency of urban agglomerations: Spatial distribution of urbanization based on heterogeneity. China Popul. Resour. Environ..

[68] Melo P.C., Graham D.J., Noland R.B. (2009). A meta-analysis of estimates of urban agglomeration economies. Reg. Sci. Urban Econ..

[69] Yu B., Su Y. (2022). How does land finance affect land use efficiency? Dynamic space Durbin model test based on the perspective of scale and technology. Geogr. Res..

[70] Liu Y., Li S., Chen Z. (2017). Polycentric Development and Its Effect on Regional Income Disparity. China Ind. Econ..

[71] Chen X., Qiu B. (2021). Polycentric spatial structure and labor income: Evidence from China’s industrial enterprises. Nankai Econ. Stud..

[72] Liu S., Hu A. (2010). Test on the Externality of Infrastructure in China: 1988–2007. Econ. Res. J..

[73] Han S., Miao C. (2022). Does a Polycentric Spatial Structure Help to Reduce Industry Emissions?. Int. J. Environ. Res. Public Health.

[74] Zou Y., Lu Y., Cheng Y. (2019). The impact of polycentric development on regional gap of energy efficiency: A Chinese provincial perspective. J. Clean. Prod..

[75] Chen X., Chen X., Song M. (2021). Polycentric agglomeration, market integration and green economic efficiency. Struct. Chang. Econ. Dyn..

[76] Tang C., Dou J. (2022). Exploring the Polycentric Structure and Driving Mechanism of Urban Regions from the Perspective of Innovation Network. Front. Phys..

[77] Odell H., Navarro-Lopez E.M., Pinto N., Deas I. (2022). Detecting shifts in metropolitan structure: A spatial network perspective. Environ. Plan. B-Urban Anal. City Sci..

[78] Lu X., Wang M., Tang Y. (2021). The Spatial Changes of Transportation Infrastructure and Its Threshold Effects on Urban Land Use Efficiency: Evidence from China. Land.

